# Cardiac MRI in patients with COVID-19 infection

**DOI:** 10.1007/s00330-022-09325-x

**Published:** 2022-12-13

**Authors:** Emad H. Abdeldayem, Basant M. Raief Mosaad, Aya Yassin, Ahmed S. Abdelrahman

**Affiliations:** Department of Radio-Diagnosis, Faculty of Medicine, Ain Shams University, Cairo, Egypt

**Keywords:** Coronavirus, Magnetic resonance imaging, Myocarditis, Takotsubo cardiomyopathy, Acute coronary syndrome

## Abstract

**Objective:**

COVID-19 infection is a systemic disease with various cardiovascular symptoms and complications. Cardiac MRI with late gadolinium enhancement is the modality of choice for the assessment of myocardial involvement. T1 and T2 mapping can increase diagnostic accuracy and improve further management. Our study aimed to evaluate the different aspects of myocardial damage in cases of COVID-19 infection using cardiac MRI.

**Methods:**

This descriptive retrospective study included 86 cases, with a history of COVID-19 infection confirmed by positive RT-PCR, who met the inclusion criteria. Patients had progressive chest pain or dyspnoea with a suspected underlying cardiac cause, either by an abnormal electrocardiogram or elevated troponin levels. Cardiac MRI was performed with late contrast-enhanced (LGE) imaging, followed by T1 and T2 mapping.

**Results:**

Twenty-four patients have elevated hsTnT with a median hsTnT value of 133 ng/L (IQR: 102 to 159 ng/L); normal value < 14 ng/L. Other sixty-two patients showed elevated hsTnI with a median hsTnI value of 1637 ng/L (IQR: 1340 to 2540 ng/L); normal value < 40 ng/L. CMR showed 52 patients with acute myocarditis, 23 with Takotsubo cardiomyopathy, and 11 with myocardial infarction. Invasive coronary angiography was performed only in selected patients.

**Conclusion:**

Different COVID-19-related cardiac injuries may cause similar clinical symptoms. Cardiac MRI is the modality of choice to differentiate between the different types of myocardial injury such as Takotsubo cardiomyopathy and infection-related cardiomyopathy or even acute coronary syndrome secondary to vasculitis or oxygen-demand mismatch.

**Key Points:**

• *It is essential to detect early COVID-related cardiac injury using different cardiac biomarkers and cardiac imaging, as it has a significant impact on patient management and outcome.*

• *Cardiac MRI is the modality of choice to differentiate between the different aspects of COVID-related myocardial injury.*

## Introduction

Coronavirus 2019 (COVID-19) was declared a global pandemic by the World Health Organization on March 11, 2020. COVID-19 infection is a multi-systemic disease with various manifestations on the different organs and tissue due to the direct effect of the virus or inflammatory mediators especially the interleukin-6 (IL-6) [1]. A variety of conditions can affect the myocardium and may have similar clinical presentations including ECG changes, elevated cardiac biomarkers, and impaired cardiac function [2]. Several viruses, like adenoviruses and enteroviruses, are implicated in the onset of myocarditis. Cardiac injury has been recognized as a complication of COVID-19 infection [3]. The mechanism of COVID-19-related myocarditis could be an immune-mediated response or a direct viral injury with consequent cardiac damage [4].

Myocarditis is an inflammatory process of the cardiac muscle, characterized by inflammatory infiltrates, interstitial edema, and myocardial injury with no evidence of obstructive coronary artery disease [5]. Some acute myocarditis COVID-like syndrome cases were reported in 2020 [6]. COVID-19 infection has also been occasionally associated with Kawasaki-like vasculitis and systemic arterial and venous thromboembolism, as well as other vascular injuries [7, 8].

Takotsubo cardiomyopathy (TCM) is a significant disease entity that differs from acute myocardial infarction. It is presented by transient hypokinesia of the left ventricular (LV) apex and is associated with emotional or physical stress, more often in elderly females. Wall motion abnormality of the LV apex is generally transient and resolves within a few days to several weeks. The prognosis was initially thought to be benign, but subsequent studies have demonstrated that short-term and long-term mortality was higher than expected. Although the pathophysiology of TCM is not clearly understood, coronary spasm, catecholamine toxicity, and myocarditis might contribute to its pathogenesis [9]. The increased incidence of TCM within the general population was noted by a large cohort study performed at the Cleveland Clinic [10]. TCM is caused by catecholamine surge, which is also observed in COVID-19 disease due to the cytokine storm. There is an association between TCM and COVID-19 reeling from the adverse psychosocial effects of the pandemic and in COVID-19 patients [11].

Generally, the clinical presentation of myocarditis varies from mild symptoms, such as fatigue and dyspnoea, to a fulminant course with heart failure and cardiogenic shock [12]. Imaging plays a crucial role in the characterization of different aspects of acute myocardial injury and enables a definite diagnosis and early treatment. Cardiac MRI (CMRI) is the reference standard for assessing patients with suspected myocarditis. CMRI provides detailed anatomical visualization, quantitative accuracy, and interobserver consistency with volumetric and functional assessment [13, 14].

T1 and T2 mapping techniques have been explored in pursuit of more quantitative and objective markers of inflammation in myocarditis to increase diagnostic accuracy. Their values are being recognized as not only robust biomarkers for the diagnosis of cardiomyopathies but also predictive factors for treatment monitoring and prognosis [15, 16].

Our study aimed to characterize the different aspects of myocardial injury in patients with COVID-19 infection using cardiac biomarkers and cardiac imaging with special emphasis on cardiac MRI as the modality of choice to differentiate between the different aspects of COVID-related myocardial injury.

## Materials and methods

### Patient population

This descriptive retrospective study included 123 patients examined between 1 March 2020 and 1 June 2021, referred to our institute for cardiac MRI. The inclusion criteria were a history of COVID-19 infection confirmed by PCR, progressive chest pain or dyspnea for 4–10 days, with a suspected underlying cardiac cause as revealed by one or more cardiac investigations, including echocardiography, troponin level, and ECG changes within 14 days from the onset of the symptoms. The exclusion criteria were one or more cardiovascular risk factors (*n* = 22) as diabetes, hypertension, dyslipidemia, and smoking, no available PCR for COVID-19 (*n* = 5), previous conventional coronary angiography including catheter balloon or percutaneous coronary intervention (PCI) (*n* = 8), one due to severe renal disease (eGFR < 30 mL/min/1.73 m^2^), and one due to contrast sensitivity.

A radiologist with 12 years of experience in cardiac imaging was responsible for interpreting the cardiac MRI images in this study. He was blinded to the patient outcome data and the other cardiac investigations, including echocardiography, ECG, laboratory profile, and conventional coronary angiography in the selected cases. The radiologist analyzed the images regarding all pulse sequences, including the black and white blood images, cine images, and late gadolinium enhancement. He calculated the functional analysis, including the ejection fraction, left ventricle end-diastolic volume, regional wall motion abnormality, and the T1 and T2 mapping of the 17 cardiac segments according to the American Heart Association guidelines (AHA).

Three cardiologists with at least 10 years of experience participated in this study. Each was responsible for one aspect of the cardiac evaluation to avoid interobserver bias. The first cardiologist was responsible for the clinical history, physical examination, and assessment of the laboratory findings. The second was responsible for performing echocardiography and interpreting the ECG findings. The third performed conventional coronary angiography in selected patients with either combined echocardiography and ECG abnormalities and/or signs of ischemic insult in cardiac MRI. All collected data were discussed by the three cardiologists for further management.

### Echocardiography and ECG

All cases underwent echocardiography examination as the first line of imaging to detect any abnormal cardiac findings with particular emphasis on regional wall motion abnormality, calculation of the LV function, and detection of other cardiac pathology. ECG was performed for all patients to detect any abnormality.

### High-sensitivity cardiac troponin T and high-sensitivity cardiac troponin I levels

These were performed for all 86 patients to detect any myocardial involvement as they are specific markers for myocardial injury.

### Cardiac MRI examination

The conventional CMR examination and additional T1 and T2 mapping were performed within seven days of troponin rise and the onset of cardiac symptoms (median of 4 days [IQR: 3 to 5 days]) using a 1.5-Tesla scanner (Siemens Aera, Siemens Healthcare).

#### A detailed description of the cardiac MRI technique followed by T1 and T2 mapping (as shown in Table 1)

- Patient position: The patients were asked to lie on the MRI table in the supine position. The ECG electrodes were placed after cleaning the skin, and the patient was monitored for heart rate and blood pressure.
- A multi-phase array coil (16 channels) required for parallel imaging was used. The examination was both ECG and respiratory gating.
- Image acquisition was initiated with localiser images in three orthogonal planes. Black-blood fast spin echo (FSE) sequences were taken in axial and coronal views. The white blood pool (GRE) sequences with true FISP were taken in the vertical long axis (VLA), short-axis (SA), and four-chamber (4 CH) views. T2-weighted-STIR images using a triple-IR black-blood turbo-spin echo pulse sequence were taken in the SA and 4 CH views. This procedure was followed by late gadolinium enhancement using a contrast-enhanced-inversion recovery sequence after 10 min from contrast injection ((0.1 mmol/kg body weight using I.V. gadobutrol (Gadovist, Bayer Schering Pharma)). Imaging of the left ventricle was performed from base to apex, and the acquisition was performed in SA and 4 CH views. Cine and LGE images were acquired during end-expiration breath-hold. Finally, modified Look-Locker inversion recovery pulse sequence (MOLLI) for T1 and T2 mapping data were acquired in basal, mid-ventricular, and apical short-axis planes before and 15 min post-contrast media injection, immediately after performing the late gadolinium enhancement [17]. Single-shot SSFP images were acquired at different inversion times (pattern 3-3-5) to generate a pixel-wise myocardial T1 and T2 map.
- Cardiac MRI image analysis:
- Multiple parameters were measured: End-diastolic and end-systolic left ventricle volumes, ejection fraction, stroke volume, and left ventricle mass for assessment of the ventricular function.
- Anatomical variants or pathologies within the cardiac chambers were assessed (e.g., masses or thrombi).
- Wall motion of the left ventricle was assessed at each myocardial segment: normal, hypokinetic, akinetic, or dyskinetic.
- Any myocardial edema on T2-weighted-STIR images were detected using the T2 signal intensity (SI) ratio by dividing the SI of the myocardium by the SI of skeletal muscle in the same slice. A hypointense core within a high SI area was included in the edematous volume. We selected the anterior chest muscles, including the major and minor pectoral muscles, as a reference range for SI of skeletal muscle.
- Any late gadolinium enhancement was detected throughout the 17 heart segments based on the 17-segment model recommended by the AHA.
- Pixel-wise illustration of the absolute T1 relaxation times on a map was performed, and color-coded maps were obtained. The ROI was then placed over the 17 heart segments in both pre- and post-contrast images according to the AHA. Six basal segments, six mid-ventricular segments, four apical segments, and cardiac apex in addition to the blood were used to obtain the pre- and the post-contrast T1 and T2 values in each of these segments.
- The diagnosis of acute myocarditis was based on updated 2018 Lake Louise criteria and CMR findings, including T1 and T2 myocardial markers.

**Table 1:** Imaging parameters for CMR examination

Sequence name	Planes of acquisition	Parameters	
Black blood [fast-spin echo (FSE) sequence]	Axial and coronal	TR ms	1300
		TE ms	35
		Field of view (FOV) mm^2^	75.5 × 38
		Section thickness (ST) 3 mm	3
White blood pool (GRE sequences): true FISP	Vertical long-axis (VLA), short-axis (SA), and four-chamber (4 CH) views.	TR ms	3.2
		TE ms	1.07
		Flip angle	65°
		Matrix	256 × 128
		Bandwidth Hz	31.25
		Slice thickness mm	6 mm, with 4 mm interslice gaps (to make a total of 10 mm)
		Field of view ranging in mm	340–400 mm
		Voxel size	1.7 × 1.7 × 10
T2-weighted STIR images using a triple-IR black-blood turbo-spin echo pulse sequence	short-axis (SA) and four-chamber (4 CH) views.	Inversion time (TI) ms	180
		Bandwidth Hz	504
		TE ms	100
Late gadolinium enhancement with inversion recovery	Imaging of the left ventricle was done from base to apex and the acquisition was performed in SA and 4 CH views	Slice thickness mm	6 mm, with 4 mm interslice gaps (to make a total of 10 mm)
		Inversion time ms	550
Modified Look-Locker Inversion recovery pulse sequence: for T1 and T2 mapping.	Imaging in basal, mid-ventricular, and apical short-axis planes.	TR ms	2.6–2.7
		TE ms	1.0–1.1
		Flip angle	35°
		FOV mm^2^	270 × 360
		matrix	156 × 208 to 168 × 224
		Slice thickness mm	6 mm, with 4 mm interslice gaps (to make a total of 10 mm)
		Linear phase-encoding ordering with minimum TI ms	91 ms

### Invasive coronary angiography

**Fig. 1:**
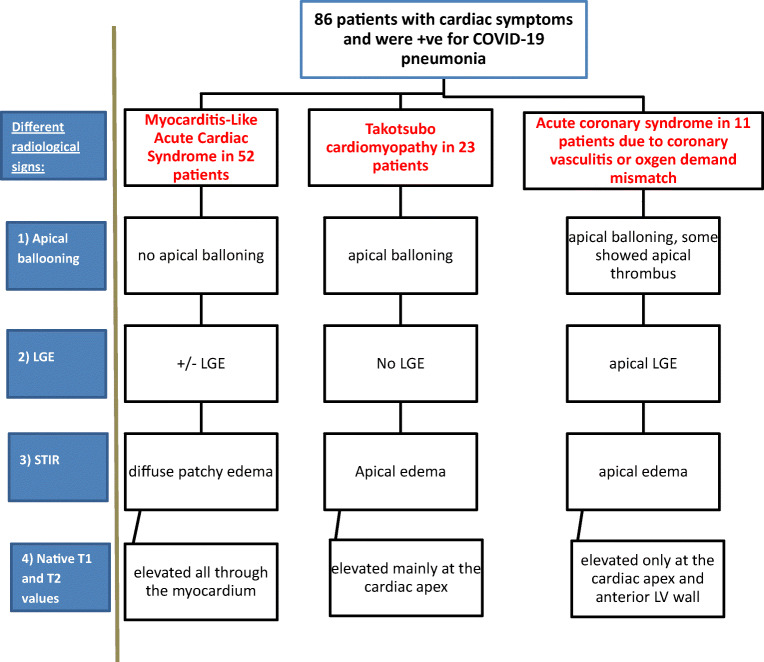
Detection of the different radiological signs using the cardiac MRI sequences for characterization of the cardiac complications in patients with COVID-19 infection

This was performed on 14 patients with combined echocardiographic and ECG abnormalities and/or signs of ischemic insults in cardiac MRI to detect the presence of any obstructive coronary artery disease (Fig. 1).

## Results

### Patient population

Our study included 86 patients (*n* = 86; 45 females and 41 males; 36 ± 7 years) who presented with a history of progressive chest pain (*n* = 68, 79.1%) and dyspnoea (*n* = 18, 20.9%) with recent symptoms of COVID-19 pneumonia, confirmed by positive PCR.

### Echocardiography findings

Regional wall motion abnormality was found in 51 of 86 patients.

### ECG findings

There was an abnormal ECG finding in the form of ST-segment elevation in 17 patients, while 15 patients had ST depression, and the remaining patients had no remarkable ECG findings.

### High-sensitivity cardiac troponin T and high-sensitivity cardiac troponin I levels

Twenty-four patients showed elevated cardiac troponin (hsTn), with a median hsTn T value of 133 ng/L (IQR: 102 to 159 ng/L); normal value < 14 ng/L. The other patients (*n* = 62) showed elevated cardiac troponin I (hsTn I) with a median hsTn I value of 1637 ng/L (IQR: 1340 to 2540 ng/L); normal value < 40 ng/L. None of the patients showed simultaneously elevated cardiac troponin (hsTn) T and troponin (hsTn) I.

#### Cardiac MRI examination (Figs. 2 and 3)

The MRI findings revealed cardiac injury in one of three patterns: (i) Takotsubo-like, (ii) myocarditis-like, and (iii) ischemic-like.

I. *The first group (n = 23 patients, 13 females and 10 males) with Takotsubo-like imaging features:* Cine images revealed normal left ventricle end-diastolic volume (median 69 mL/m^2^ (IQR: 62 to 78 mL/m^2^); normal value < 90 mL/m^2^). The images showed ballooning of the cardiac apex, with severe depression of systolic function (ejection fraction < 40%) and no LGE. The T2-weighted-STIR images showed myocardial edema, which was generally diffuse and transmural, involving mainly the regions with RWMA. A T2 SI ratio (ratio of SI between the myocardium and skeletal muscle) greater than or equal to 1.9 was considered diagnostic of myocardial edema. T1 and T2 mapping values were elevated, with a median of 1165 ms (IQR: 1131to 2101 ms); normal value < 1045 ms for T1 mapping and a median of 66 ms (IQR: 59 to 69 ms); normal value < 50 ms for T2 mapping. Both T1 and T2 mapping showed an increasing gradient from the base to the apex. This pattern suggests that myocardial inflammation is global but affects the apical segments predominantly. All these patients were diagnosed with Takotsubo cardiomyopathy and experienced complete recovery of the LV function within 4–6 weeks on follow-up echocardiography examination (Fig. 4).
II. *The second group (n = 52 patients, 26 females and 26 males) with myocarditis-like imaging features:*
  The 2018 Lake Louise criteria were applied for the diagnosis of myocarditis. The two main 2018 Lake Louise criteria are T1-based and T2-based. The T1-based criterion was positive if there was an increase in native T1 relaxation times, an increase in extracellular volume (ECV), or a positive LGE. The T2-based criterion is positive in cases of increased T2 relaxation times, regional high T2 signal intensities on T2-weighted images, or an increased global T2 SI ratio [18].
  There was a positive T1-based criterion in the form of increased native-T1 mapping (median 1162 ms [IQR: 1128 to 1199 ms]; normal value < 1045 ms) in all 52 patients. There was also patchy mesocardial late gadolinium enhancement, which was present in 29 patients, while the remaining 23 cases had no LGE.
  There was a positive T2-based criterion in the form of increased T2 mapping (median 64 ms [IQR: 58 to 69 ms]; normal value < 50 ms) in all patients. There was also myocardial edema, with regional or global hyperintensity, which was not limited to vascular territory. There was predominant sub-epicardial or mid-myocardial distribution, with increased myocardial-to-skeletal muscle intensity ratio on T2-weighted-STIR images (median T2-ratio: 2.5 [IQR: 2.4 to 2.6]; normal value < 1.9).
  On functional and cine images, 31 patients had preserved ejection fraction (> 55%) with no regional or global wall motion abnormality, while 21 had mildly impaired ejection fraction (40 to 55%), 13 with regional wall motion abnormalities, and eight with global wall motion abnormality in the form of hypokinetic LV wall (Fig. 5).
III. *The third group (n = 11 patients, six females and five males) with ischemic-like imaging features:* CMR scans showed increased left ventricle end-diastolic volume (median 98 mL/m^2^ [IQR: 97 to 99 mL/m^2^]; normal value < 90 mL/m^2^). Severe affection of systolic function (ejection fraction < 40%) was also noted with akinetic apex and apical thrombus in four cases. There was apical edema on T2-weighted STIR and transmural LGE of the cardiac apex keeping with myocardial scarring. There was also an increased native T2 value (median T2-ratio: 2.5 [IQR: 2.5 to 2.7]) and increased native-T1 value (median 1172 ms [IQR: 1170 to 1174 ms]) (Fig. 6).

**Fig. 2:**
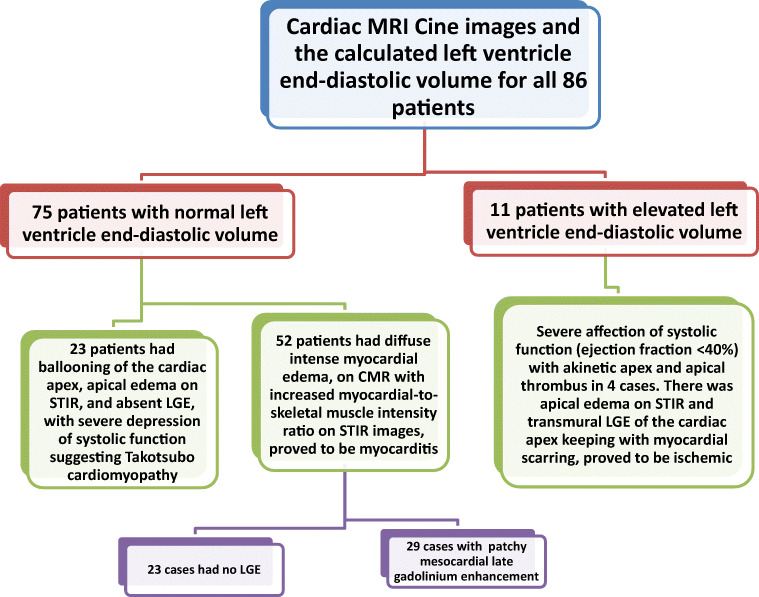
Algorithm representing the spectrum of analysis of cardiac MRI finding regarding the end-diastolic volume and approach through the abnormal finding to differentiate between cardiac complications in patients with COVID-19 infection

**Fig. 3:**
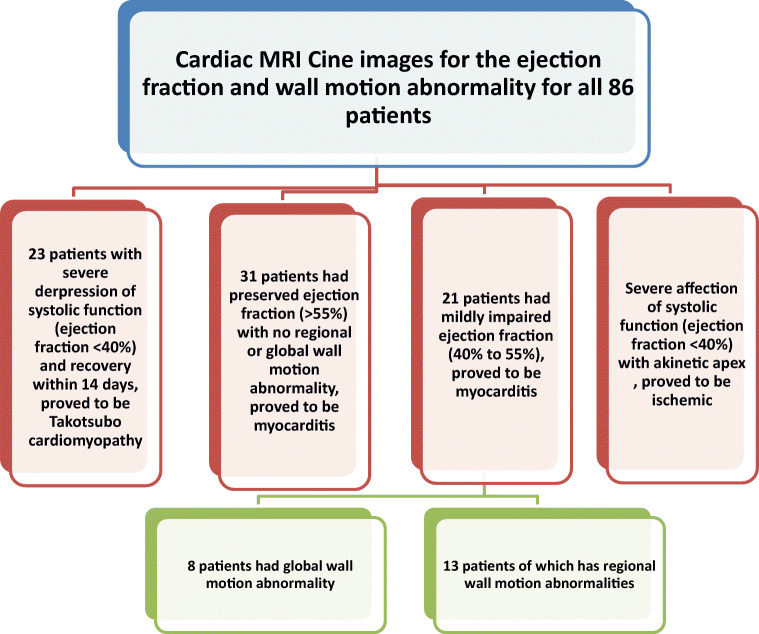
Algorithm representing the spectrum of analysis of cardiac MRI finding regarding the ejection fraction and wall motion abnormality to differentiate between cardiac complications in patients with COVID-19 infection

**Fig. 4:**
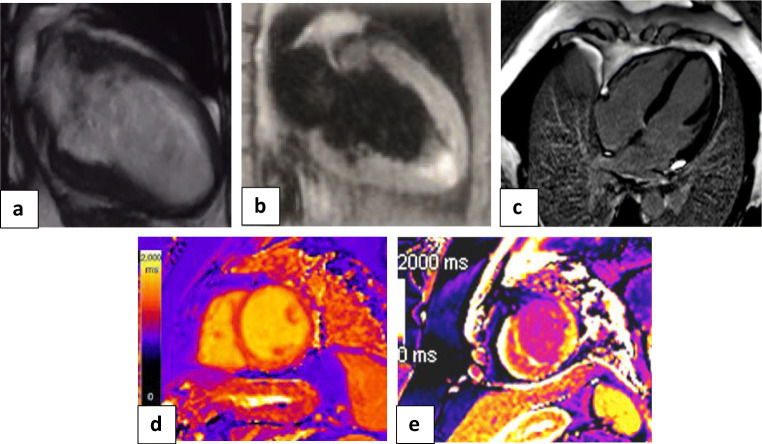
Cardiac MRI: **a** white blood image 2-chamber vertical long-axis view showing ballooning of the cardiac apex. **b** T2-weighted-STIR image 2-chamber view showing interstitial edema of the cardiac apex. **c** IR post-contrast image 4-chamber view showing no appreciable LGE of the cardiac apex. **d**, **e** T2 and T1 cardiac MRI mapping sequence in two-chamber views respectively showing intense interstitial myocardial edema (native T2 = 64 ms) and elevated T1 value =1178 ms. Conventional coronary angiography of LAD confirmed normal appearance with no stenosis or occlusion. The final diagnosis was Takotsubo-like cardiomyopathy (as a part of acute myocarditis COVID-like syndrome (AMCovS)

**Fig. 5:**
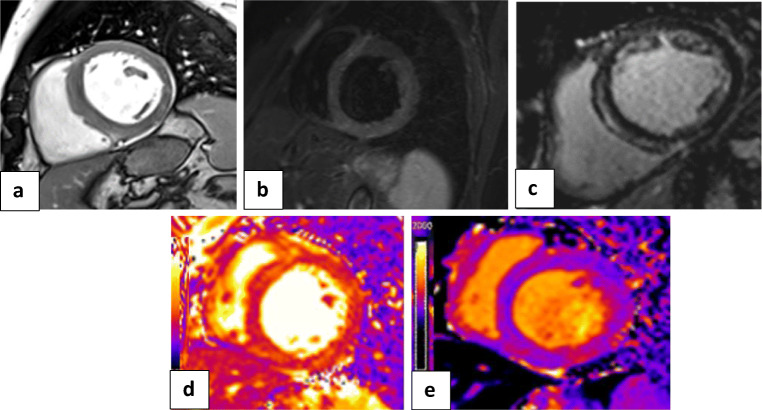
Cardiac MRI: **a** white blood image 2-chamber short-axis view showing preserved shape and configuration of the left ventricle. **b** T2-weighted-STIR image 2-chamber short axis showing faint patchy interstitial edema of the myocardium. **c** IR post-contrast image 2-chamber view showing multiple patchy mid-myocardial enhancing zones of the late gadolinium enhancement, more evident at basal septal and inferior walls. **d**, **e** T2 and T1 cardiac MRI mapping sequence in two-chamber view respectively showing intense interstitial myocardial edema with native T2 = 67 ms and elevated T1 value = 1154 ms. Conventional coronary angiography of LAD confirmed normal appearance with no stenosis or occlusion. The final diagnosis was acute myocarditis COVID-like syndrome (AMCovS)

**Fig. 6:**
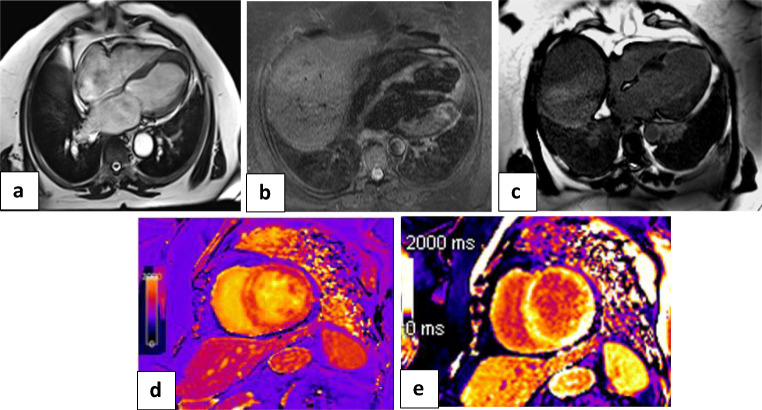
Cardiac MRI: **a** White blood image 4-chamber view showing ballooning and thinning out of the cardiac apex with apical thrombus. **b** T2-weighted-STIR image 4-chamber view showing faint interstitial edema of the cardiac apex and apical cardiac segments. **c** IR post-contrast image 4-chamber view showing sub-endocardial / trans-mural enhancing zones of the late gadolinium enhancement along with the LAD territory. **d**, **e** T2 and T1 cardiac MRI mapping sequence in two-chamber view respectively showing mild interstitial myocardial edema (native T2 = 58 ms) and elevated T1 value = 1193 ms. Conventional coronary angiography of LAD confirmed tapering and narrowing of distal LAD. The LCX and RCA were normal. The final diagnosis was acute coronary syndrome due to coronary vasculitis or oxygen demand mismatch

Invasive coronary angiography was performed for 14 patients with electrocardiography abnormalities or a pattern of myocardial ischemia on cardiac MRI to detect the presence of obstructive coronary artery disease. Three patients had a normal conventional coronary angiogram, while 11 with akinetic apex and transmural LGE on CMR had attenuated distal LAD confirming the presence of an ischemic insult.

No pericardial effusion or abnormal pericardial thickening was detected in the 86 patients.

Follow-up occurred after 4 weeks, focusing on the clinical presentation (chest pain and dyspnoea), high-sensitivity troponin level, echocardiography, and ECG changes. Seventy-five patients diagnosed with myocarditis-like and Takotsubo-like findings were discharged with the resolution of chest pain and near-total improvement of the dyspnoea. There was normalization of the ECG changes and cardiac markers and improvement of the LV function as revealed by echocardiography. The 11 patients who presented with associated ischemic changes still had dyspnoea that showed no significant improvement. Chest pain almost disappeared with regression of the ECG changes and high-sensitivity troponin level. Echocardiography still showed increased LV end-diastolic volume and severe affection of systolic function (ejection fraction < 40%).

## Discussion

The previous SARS epidemics and the ongoing COVID-19 pandemic have produced multiple theories regarding cardiac damage. One of these is a systemic inflammatory response involving the release of high levels of cytokines that result in damage to the vascular endothelium and cardiac myocytes [19–22]. Another theory regarding myocardial damage proposes that the interaction of SARS-CoV-2 with ACE2 causes changes to the ACE2 pathways, leading to injury of the cardiac muscle and endothelial cells [23]. Furthermore, plaque rupture leading to acute coronary syndrome can result from systemic inflammation. Other possible mechanisms include cardiotoxic side effects of corticosteroids, antiviral medications, and immunological agents [23]. Moreover, electrolyte disturbances can occur in any critical illness and can trigger arrhythmias, especially in patients with an underlying cardiovascular disease [24].

COVID-19 infection may trigger new cardiac pathologies or exacerbate underlying cardiovascular diseases due to the associated inflammatory process. There was a small number of cases have been reported with AMCovS showing an absence of or very minimal LGE. Thus, may suggest an indirect mechanism causing myocardial inflammation with an absence of myocardial scarring [25].

Antonio Esposito et al (2020) reported the first series of patients with AMCovS, including 10 patients with clinical suspicion of myocarditis referred for CMR examination. The CMR revealed acute myocarditis in eight patients and Takotsubo cardiomyopathy in two. There was diffuse myocardial edema in T2-weighted-STIR images and no appreciable LGE, suggesting an indirect mechanism causing myocardial inflammation [26].

The present study included 86 patients who presented with a history of progressive chest pain or dyspnoea, with suspected underlying cardiac cause. The patients had recent symptoms of COVID-19 pneumonia confirmed by positive PCR. All patients performed electro-echocardiography and troponin level assay. Conventional CMR examinations and additional T1 and T2 mapping were performed within seven days of troponin rise and the onset of cardiac symptoms to detect and characterise any myocardial injury.

We were able to explain the proposed mechanisms of cardiac injury through one of three disorders: Takotsubo cardiomyopathy, myocarditis-like acute cardiac syndrome, and ischemia due to vasculitis or other oxygen supply-demand mismatch.

Takotsubo cardiomyopathy is non-ischemic cardiomyopathy, more often seen in postmenopausal elderly women and characterized by the ballooning of the cardiac apex. Its clinical manifestations are similar to those of acute coronary syndrome; however, Takotsubo cardiomyopathy is usually preceded by emotional stress and has spontaneous recovery [27, 28]. In our study, there was a severe depression of systolic function and no LGE to suggest scarring or fibrosis. Also, T1 and T2 mapping values were high, showing an increasing gradient from the base to the apex. This pattern suggests that myocardial inflammation predominantly affects the apical segments. These patients experienced complete recovery of the LV function within 4–6 weeks on follow-up as revealed by echocardiography examination.

Myocarditis is a non-ischemic inflammatory disease of the myocardium that can be triggered by variable events, including viral infection and toxins. Cardiac MRI is the modality of choice for the diagnosis of myocarditis, with typical findings of focal or diffuse myocardial edema and myocardial damage, including the presence of late gadolinium enhancement [29–32]. In our study, we applied the updated 2018 Lake Louise criteria for diagnosis of myocarditis (i.e., T1-based criterion and T2-based criterion). The systolic function was either preserved or mildly impaired. Cine images showed regional or global wall motion abnormality in 21 patients. The pattern of myocarditis, like acute coronary syndrome, was similar to other types of myocarditis in the form of regional or global myocardial edema, not limited to vascular territory. There was also patchy mesocardial late gadolinium enhancement in 29 patients, with a predilection for lateral wall and basal septal enhancement. All patients with myocarditis-like acute coronary syndrome had complete recovery of the LV function on follow-up examination by echocardiography.

The third group with ischemic-like imaging features showed increased LV end-diastolic volume and severe affection of systolic function. There was apical edema on T2-weighted STIR and transmural LGE of the cardiac apex keeping with myocardial scarring, in contrast to Takotsubo cardiomyopathy. Furthermore, there were increased native T1 and T2 values. There was also attenuated distal LAD by invasive coronary angiography, confirming the presence of an ischemic insult.

All patients in the study had elevated cardiac troponin levels. The high sensitivity and specificity of cardiac troponin levels significantly impact early diagnosis and risk stratification in patients presenting with chest pain. Detection of cardiac troponin is not pathognomonic for acute coronary syndrome but rather suggests myocardial injury of any cause and may be found in non-coronary artery-related conditions [33]. One of the proposed hypotheses of elevated troponin levels involves the exacerbation of the patient’s subclinical coronary artery disease by sepsis and oxygen supply-demand mismatch, which could precipitate ischemia resulting in type 2 myocardial infarction [34].

It was observed that patients with severe COVID-19 pneumonia can develop cutaneous vasculitis-like lesions and systemic arterial and venous thromboemboli. Kawasaki-like disease has been reported in the context of acute severe myocarditis, without overt coronary vasculitis or aneurysmal formation. There are few reported cases of demonstrable coronary vasculitis with a Kawasaki-like disease phenotype [6, 35]. In our study, 11 cases presented with myocarditis and associated ischemic changes in the LAD territory, probably due to coronary vasculitis or possibly oxygen supply-demand mismatch with the formation of type 2 infarction [36, 37].

Cardiac MRI plays a vital role in the characterization of the different aspects of myocardial injury post-COVID-19 infection and thus adds to the treatment of cardiovascular injury associated with SARS-CoV-2 infection. COVID patients with a known history of pre-existing cardiovascular disease should be closely monitored for any symptoms and signs of cardiovascular dysfunction. The American College of Cardiology (ACC) recently indicated that elderly patients may need plaque stabilizers (e.g., statins, β-blockers, ACE inhibitors, or aspirin). When an acute myocardial injury occurs, medication to improve myocardial energy metabolism can significantly protect and improve cardiac function. Thrombolysis is preferred for patients with non-ST-elevation myocardial infarction (NSTEMI) or ST-elevation myocardial infarction (STEMI) without relevant contraindications. Emergency PCI may be performed in patients with acute STEMI with hemodynamic instability and patients with life-threatening NSTEMI [37–40].

Some key points emerge from this review of myocardial injury. A high clinical suspicion should be maintained for COVID-19-associated cardiac injury for early detection of any myocardial insult using different cardiac biomarkers and cardiac imaging. Our review highlights the many proposed mechanisms of myocardial damage secondary to COVID-19 infection, but more research is required to determine whether the damage is due to direct myocardial injury by the virus, inflammatory mediated process, vasculitis, or any other mechanism.

## Study limitations

The study design was retrospective. Multi-center contributions and data from other countries will be valuable in better assessing rare but severe complications of COVID-19 pneumonia. Endo-myocardial biopsy, which is the golden standard for myocarditis, was not performed due to its invasive nature.

## Conclusion

The diagnosis of myocardial injury using clinical and imaging criteria is challenging due to its highly heterogeneous manifestations. Cardiac MRI is considered the modality of choice to differentiate between the different aspects of myocardial injury such as Takotsubo cardiomyopathy and myocarditis-like acute cardiac syndrome. Moreover, the spectrum of cardiac involvement can be extended to include acute coronary syndrome secondary to vasculitis or oxygen-demand mismatch. Early and correct diagnosis has a significant impact on the patient outcome and further management.

## Abbreviations

ACE2: Angiotensin-converting enzyme 2
ACEI: Angiotensin-converting enzyme inhibitor
AMCovS: Acute myocarditis COVID-like syndrome
CMRI: Cardiac magnetic resonance imaging
COVID: Coronavirus disease
eGFR: Estimated glomerular filtration rate
hsTnI: High-sensitivity troponin I
hsTnT: High-sensitivity troponin T
hsTn: High-sensitivity troponin
LGE: Late gadolinium-enhanced
NSTEMI: Non-ST-elevation myocardial infarction
RT-PCR: Reverse transcription-polymerase chain reaction
TCM: Takotsubo cardiomyopathy

## Declarations

### Guarantor

The scientific guarantor of this publication is Dr/ Emad Hamid Abdeldayem, MD, Associate professor of Radiology, at Ain Shams University, Cairo, Egypt.

### Statistics and biometry

No complex statistical methods were necessary for this paper.

### Methodology

descriptive retrospective study
